# Supplementary material for: Serotonin-dependent kinetics of feeding bursts underlie a graded response to food availability in C. elegans
Source: Nat Commun. 2017 Feb 1;8:14221. doi: 10.1038/ncomms14221 (PMC5296638; doi:10.1038/ncomms14221)
Supplement: Supplementary Information — Supplementary Figures and Supplementary Tables [file ncomms14221-s1.pdf]

Supplementary Figures

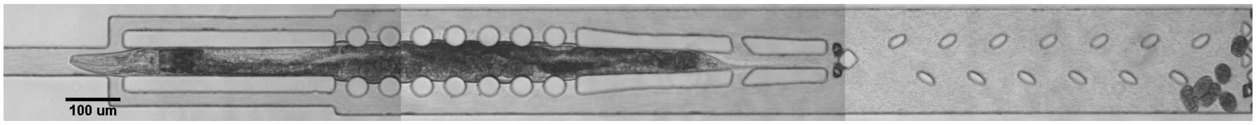

Supplementary Figure 1. A worm in the microfluidic device. Worms in the device feed on a bacterial suspension that is continuously flown through the chamber. The pillars restrain the worms from escaping while providing enough space for egg laying. Eggs are collected in an egg-collection area.

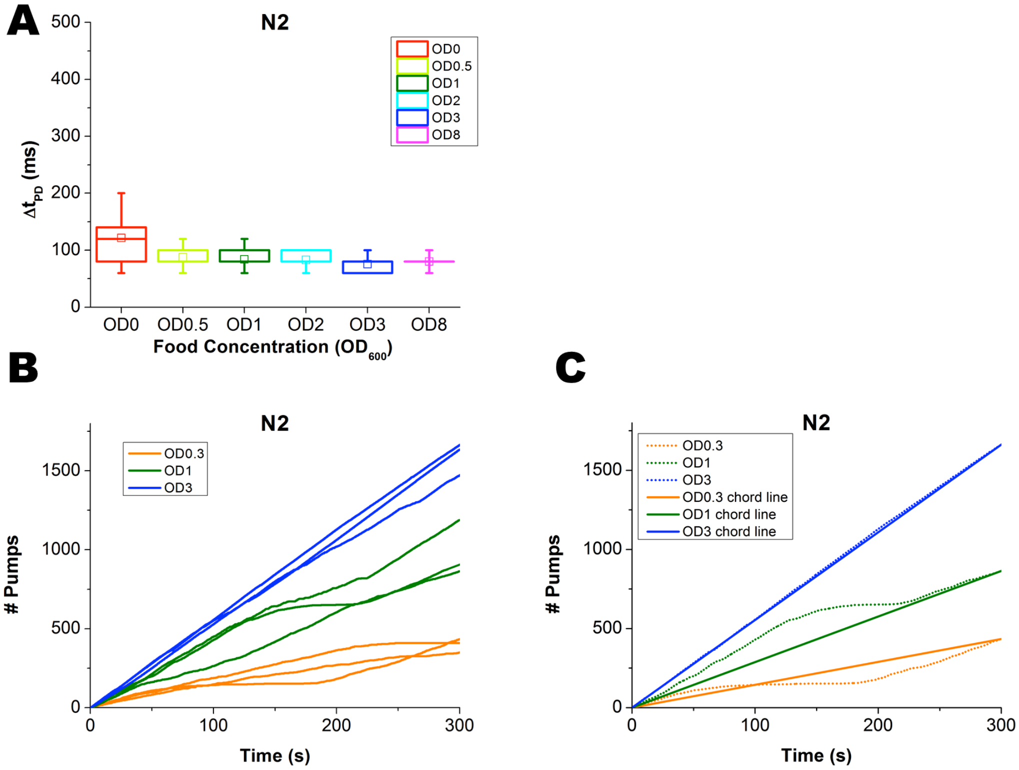

Supplementary Figure 2. Pumping dynamics depend on food concentrations. (A) Food concentration dependence of average pump duration time (Δt_{PD}). The bar graph shows average pump duration time (Δt_{PD}) with standard errors at respective food concentrations. (B) The number of pumps observed since the beginning of the experiment (time 0) for 3 different worms at each of 3 densities (OD_{600}=0.3, 1, and 3), demonstrating the reproducibility of the assay. (C) Comparison of measured pumping counts (dotted curves) with their chord line (i.e. the lines that connect the two end points of the measured curves). The slopes of these chord lines are the average pumping rates.

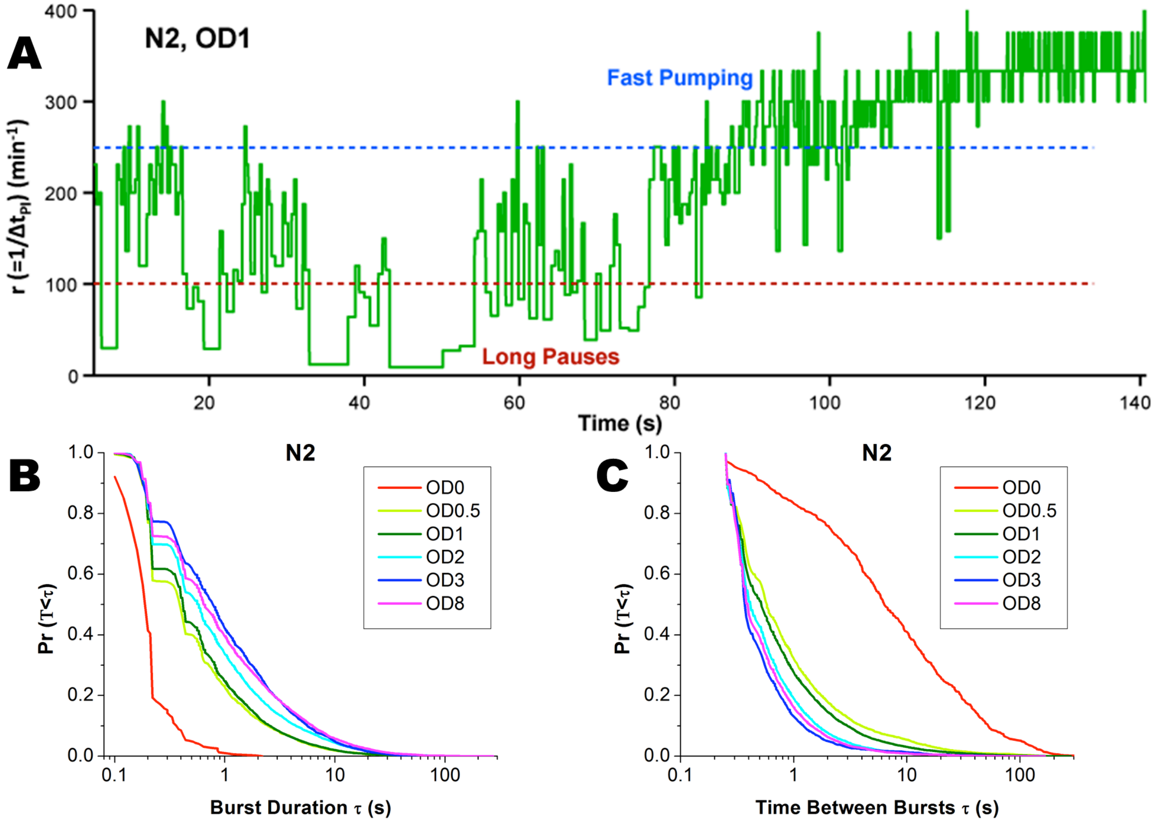

Supplementary Figure 3. Bursts of fast pumping are longer and more frequent at higher food densities. (A) A representative time series of pharyngeal pumping of a wild-type (N2) worm at OD_{600}=1. Each pumping event is indicated by its instantaneous rate, i.e. the inverse of the time interval between that pump and the next. The time series shows long pauses, fast pumping, and pumping at intermediate rates. Blue and red dashed lines indicate the thresholds for fast pumping (250 pumps/min) and long pauses (100 pumps/min), respectively. (B, C) Survival probability of burst duration (B) and the time between bursts (C) at various food concentrations.

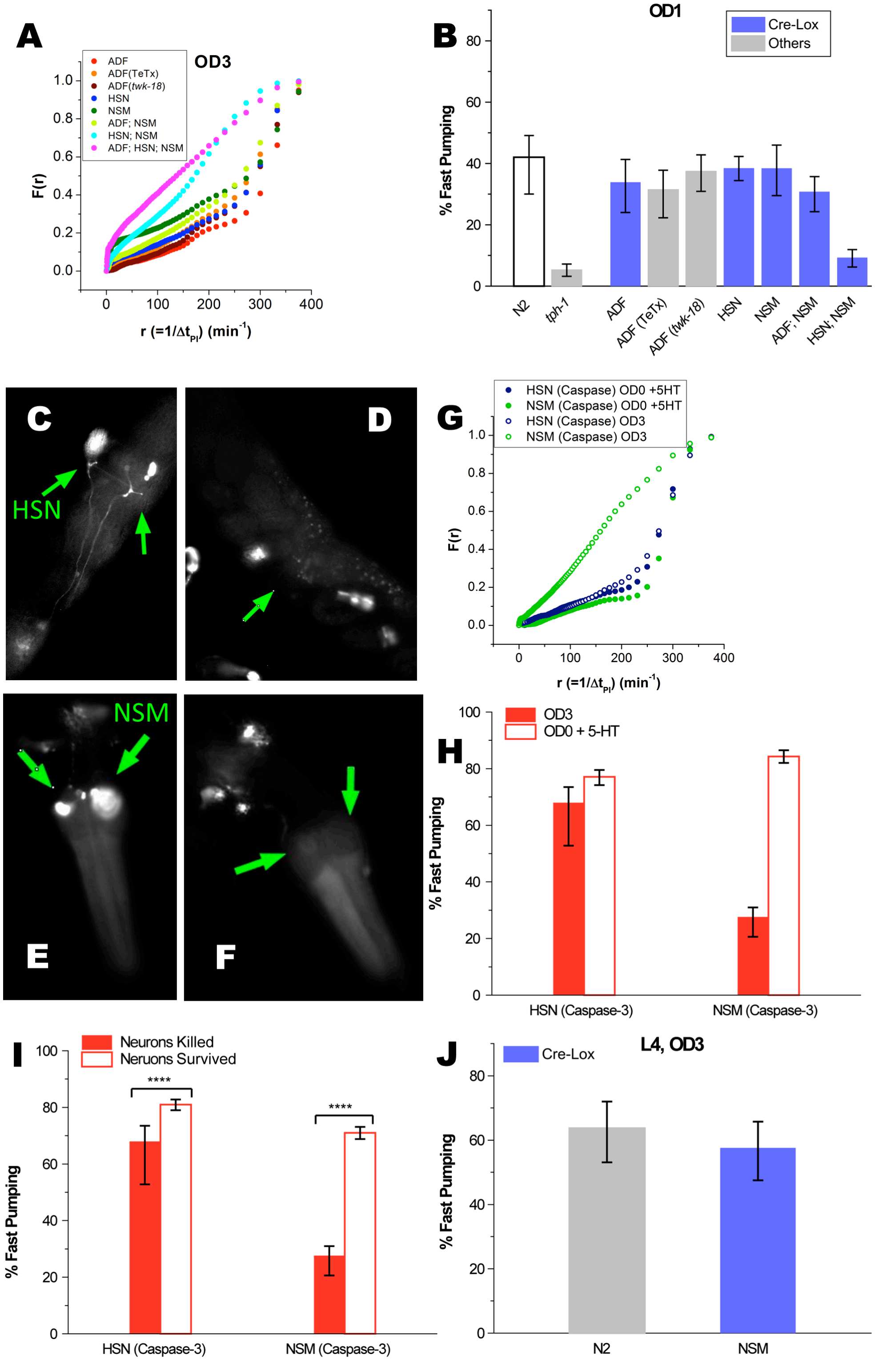
A
OD3
ADF
ADF(TeTx)
ADF(twk-18)
HSN
NSM
ADF; NSM
HSN; NSM
ADF; HSN; NSM
F(r)
r (=1/Δt_PI) (min^-1)
B
OD1
Cre-Lox
Others
% Fast Pumping
N2
tph-1
ADF
ADF (TeTx)
ADF (twk-18)
HSN
NSM
ADF; NSM
HSN; NSM
C
HSN
D
E
NSM
F
G
HSN (Caspase) OD0 +5HT
NSM (Caspase) OD0 +5HT
HSN (Caspase) OD3
NSM (Caspase) OD3
F(r)
r (=1/Δt_PI) (min^-1)
H
OD3
OD0 + 5-HT
% Fast Pumping
HSN (Caspase-3)
NSM (Caspase-3)
I
Neurons Killed
Neruons Survived
****
****
% Fast Pumping
HSN (Caspase-3)
NSM (Caspase-3)
J
Cre-Lox
L4, OD3
% Fast Pumping
N2
NSM

Supplementary Figure 4. Differential roles for the serotonergic neurons NSM and HSN in regulating fast pumping. (A) $F(r)$, the fraction of time spent in intervals longer than $1/r$, at OD_{600}=3. ADF(TeTx): strain in which the tetanus toxin is expressed from an ADF-specific promoter; ADF(*twk-18*): strain in which the activated form of potassium channel *twk*-18 is expressed from a ADF-specific promoter; All others: strains in which *tph-1* has been removed specifically in the indicated neuron(s) using the Cre/Lox approach. (B) The average fraction of time spent in fast pumping by the same worms at OD_{600}=1, compared to wild-type (N2) worms and *tph-1* mutants. Error bars as in Fig. 2. (C-F) Fluorescent images of the transcriptional reporter *tph-1p::GFP* from the HSN neurons (C,D) or the NSM neurons (E,F). Panels C and E show intact neurons, while panels D and F are images from the same region of worms in which the respective neurons were ablated by reconstituted caspase-3. (G, H) Comparison of HSN-ablated and NSM-ablated worms, at OD_{600}=3 or at 5mM exogenous 5-HT with no food. (G) $F(r)$, the fraction of time spent in intervals longer than $1/r$. (H) The average fraction of time spent in fast pumping. Error bars as in Fig. 2. (I) Comparison of the average fraction of time spent in fast pumping in worms carrying reconstituted caspase at OD_{600}=3, when the neurons were ablated (filled) and the neurons survived (empty). Error bars as in Fig. 2. Statistical significance as in Fig. 4. (J) The average fraction of time spent in fast pumping in L4 larvae. Error bars as in Fig. 2.

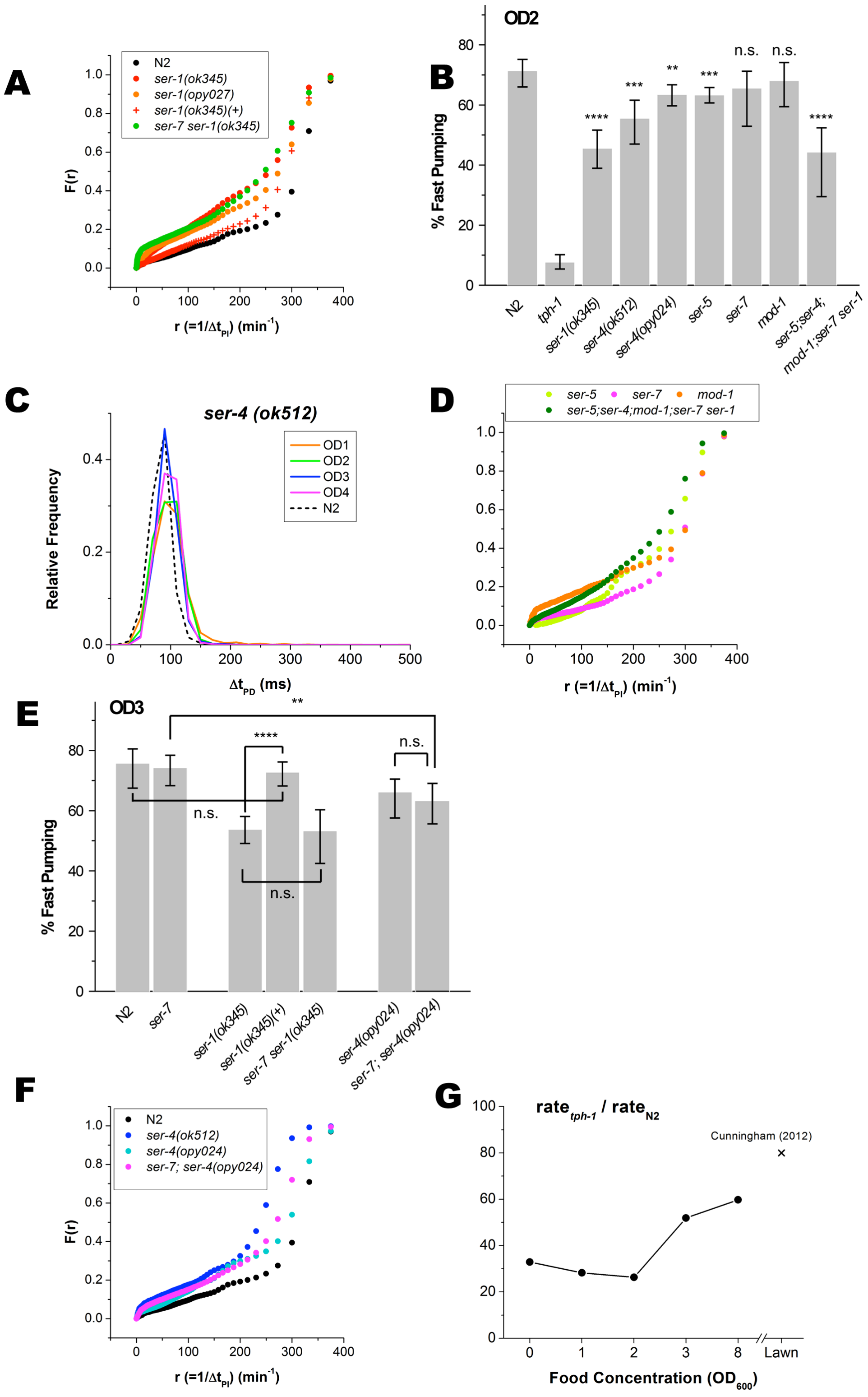
A
N2
ser-1(ok345)
ser-1(opy027)
ser-1(ok345)(+)
ser-7 ser-1(ok345)
F(r)
r (=1/Δt_PI) (min^-1)
B
OD2
% Fast Pumping
****
***
**
***
n.s.
n.s.
****
N2
tph-1
ser-1(ok345)
ser-4(ok512)
ser-4(opy024)
ser-5
ser-7
mod-1
ser-5;ser-4;
mod-1;ser-7 ser-1
C
ser-4 (ok512)
OD1
OD2
OD3
OD4
N2
Relative Frequency
Δt_PD (ms)
D
ser-5
ser-7
mod-1
ser-5;ser-4;mod-1;ser-7 ser-1
r (=1/Δt_PI) (min^-1)
E
OD3
**
****
n.s.
n.s.
n.s.
% Fast Pumping
N2
ser-7
ser-1(ok345)
ser-1(ok345)(+)
ser-7 ser-1(ok345)
ser-4(opy024)
ser-7; ser-4(opy024)
F
N2
ser-4(ok512)
ser-4(opy024)
ser-7; ser-4(opy024)
F(r)
r (=1/Δt_PI) (min^-1)
G
rate_tph-1 / rate_N2
Cunningham (2012)
Lawn
Food Concentration (OD_600)

Supplementary Figure 5. (A) $F(r)$, the fraction of time spent in intervals longer than $1/r$, at OD_{600}=3 for two mutants alleles of *ser-1*, a transgenic rescue, and the *ser-7 ser-1(ok345)* double mutant. (B) The average fraction of time spent by these worms in fast pumping at OD_{600}=2. Error bars and thresholds as in Fig. 5. Statistical significance of difference from wild-type behavior indicated as in Fig. 4. (C) Histograms of pump duration (Δt_{PD}) in *ser-4(ok512)* at various food concentrations (colored curves), compared to wild-type (N2) at OD_{600}=3 (black, dashed). (D) $F(r)$ for several serotonin receptor mutants, at OD_{600}=3. (E) The average fraction of time spent in fast pumping in worms carrying combinations of mutant alleles in serotonin receptors, at OD_{600}=3. Error bars as in Fig. 2, statistical significance as in Fig. 4. (F) $F(r)$ for two mutants alleles of *ser-4*, along with *ser-7; ser-4(opy024)* double mutant. (G) Ratio between the average pumping rate of *tph-1* worms and that of N2 worms at various food concentrations. Included for comparison is the same ratio for worms feeding on a lawn of bacteria on agar plates, as measured in Cunningham *et al.*[32].

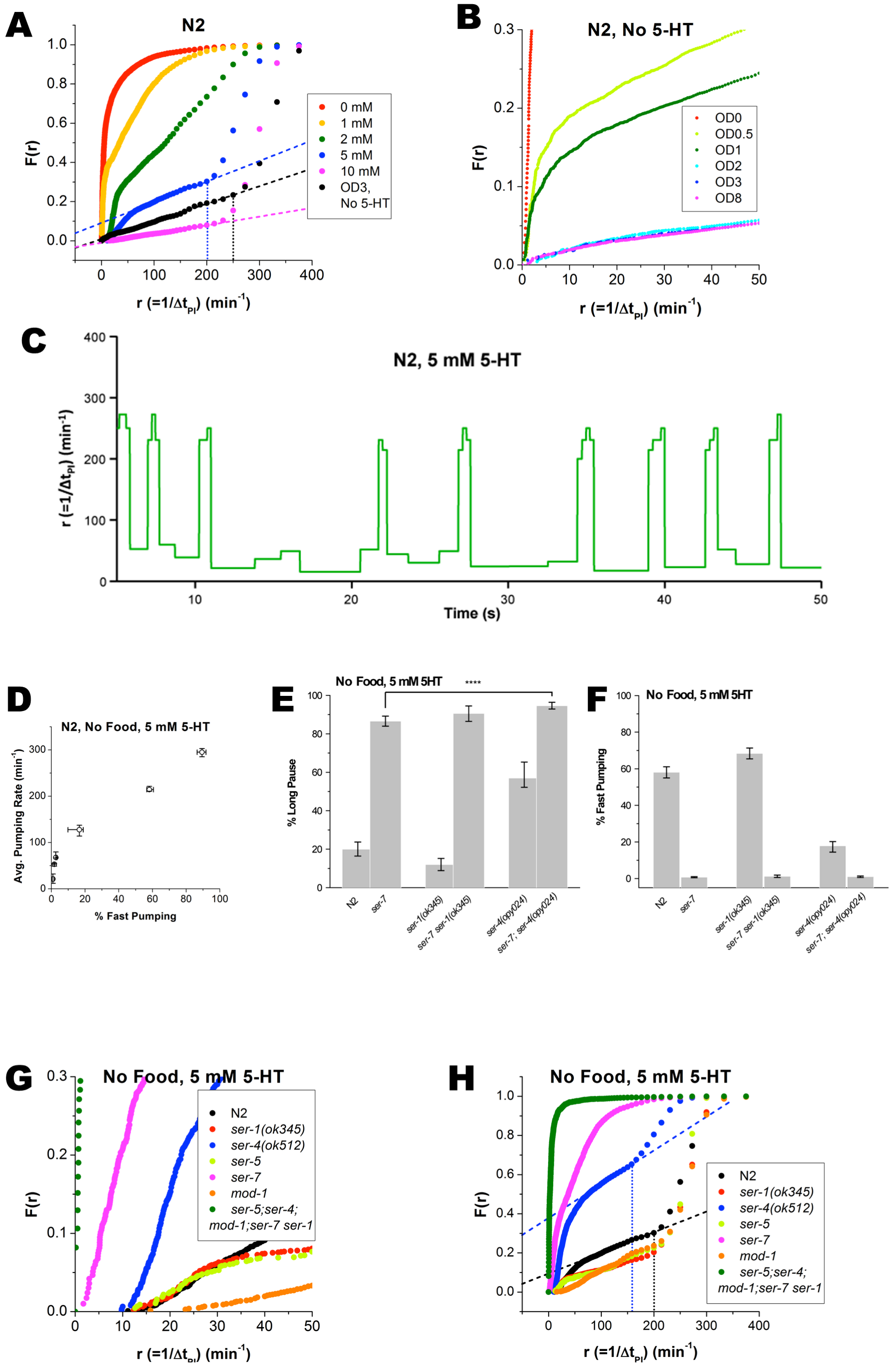
A
N2
F(r)
r (=1/Δt$_{PI}$) (min^{-1})
0 mM
1 mM
2 mM
5 mM
10 mM
OD3,
No 5-HT
B
N2, No 5-HT
F(r)
r (=1/Δt$_{PI}$) (min^{-1})
OD0
OD0.5
OD1
OD2
OD3
OD8
C
N2, 5 mM 5-HT
r (=1/Δt$_{PI}$) (min^{-1})
Time (s)
D
N2, No Food, 5 mM 5-HT
Avg. Pumping Rate (min^{-1})
% Fast Pumping
E
No Food, 5 mM 5HT
****
% Long Pause
N2
ser-7
ser-1(ok345)
ser-7 ser-1(ok345)
ser-4(opy024)
ser-7; ser-4(opy024)
F
No Food, 5 mM 5HT
% Fast Pumping
N2
ser-7
ser-1(ok345)
ser-7 ser-1(ok345)
ser-4(opy024)
ser-7; ser-4(opy024)
G
No Food, 5 mM 5-HT
F(r)
r (=1/Δt$_{PI}$) (min^{-1})
N2
ser-1(ok345)
ser-4(ok512)
ser-5
ser-7
mod-1
ser-5;ser-4;
mod-1;ser-7 ser-1
H
No Food, 5 mM 5-HT
F(r)
r (=1/Δt$_{PI}$) (min^{-1})
N2
ser-1(ok345)
ser-4(ok512)
ser-5
ser-7
mod-1
ser-5;ser-4;
mod-1;ser-7 ser-1

Supplementary Figure 6. Stimulated pumping by exogenous serotonin. (A) $F(r)$, the fraction of time spent by wild-type (N2) worms in intervals longer than $1/r$ in bacteria-free media supplemented with exogenous serotonin. For comparison, black symbols corresponds to worms feeding on bacteria at OD_{600}=3 with no serotonin added. The dotted vertical lines indicate the last point of the linear segment of $F(r)$, taken as the threshold rate for fast pumping. (B) Zoom-in on the low-rate end of **Fig. 3A** demonstrates the presence of long pauses at all food concentrations. (C) A representative time series of serotonin-induced pumping, illustrating the suppression of very long pauses. (D) Average pumping rates are plotted against the fraction of fast pumping at different concentrations of serotonin in bacteria-free media. (E, F) *ser-7 ser-1(ok345)* and *ser-7; ser-4(opy024)* double mutants compared to N2 and single mutant allele *ser-1(ok345), ser-4(opy024)* and *ser-7* in a media supplemented with 5mM 5-HT in the absence of food in (E) long pauses and (F) fast pumping. Statistical significance as in Fig. 4. (G) $F(r)$ for wild-type worms and serotonin receptor mutants in bacteria-free media with 5mM serotonin. Zoom-in on the low-rate end demonstrates the suppression of long pauses. (H) Same as in panel G. Dashed lines are the best-fit lines between 100 pumps/min and 250 pumps/min. Dotted vertical lines as in (A).

Supplementary Tables

Supplementary Table 1. Primers

Primer	Sequence (5'-to-3')
**Transgenic rescue of *ser-1***	
ser-1 F	agtcgacacatttgccaacc
ser-1 R	cagtgcttggaaggaggaga
**CRISPR: sgRNA oligos**	
ser-1 sg1 F	gagaggagaaggctggccgg gttttagagctagaaatagcaag
ser-1 sg2 F	gcacgggatgagctcacacgg gttttagagctagaaatagcaag
ser-4 sg1 F	gaaattctaatttaaggtagg gttttagagctagaaatagcaag
ser-4 sg2 F	gagccgggaattgtaaagagg gttttagagctagaaatagcaag
**CRISPR: PCR primers for homology arms (us: upstream arm; ds: downstream arm)**	
ser-1 us F	aacgacggccagtgaattcccgcgg caagttctagtagcaagttgaactag
ser-1 us R	ttataggccgcctgatgcgccgcgg gaggaaagcgaaaaaagtatg
ser-1 ds F	atagttgcaggaccactgcggccgc aacgacagaggcatcaacc
ser-1 ds R	atgattacgccaagcttgcggccgc cacactttggcacaaaactg
ser-4 us F	aacgacggccagtgaattcccgcgg ggttcaggtatactgttttcag
ser-4 us R	ttataggccgcctgatgcgccgcgg gaatatatgcttattggggg
ser-4 ds F	atagttgcaggaccactgcggccgc ggcgaacgaaagagtcgaa
ser-4 ds R	atgattacgccaagcttgcggccgc ctcacgggttactgtagtctttgg
**CRISPR: primer for PCR-verification of insertion at target site**	
ser-1 us F	gggttgtaattttgaaattccagg
ser-1 ds R	gcaccctatgatctacactttttc
ser-4 us F	cacgccgagattttcagc
ser-4 ds R	catatcggctactttcggcc
Pmyo-2 R	gtgagtcgtattacgtacgaggtacc
unc-54 F	cattcccaatataccaaacataactg

Supplementary Table 2. Experiment Details

All experiments were done in two independent biological repeats, unless noted otherwise.

Figure	Experiment Details
Fig. 2, Fig. 3	The numbers of animals used for OD_{600}=0, 0.5, 1, 2, 3, 8 are 62, 61, 113, 64, 33, 84, respectively. 5mins/animal. The experiments were repeated more than twice for the followings: 4, 5, 3, 4, 5 repeats for OD_{600}=0, 1, 2, 3, 8, respectively.
Fig. 4A	The numbers of animals used for OD_{600}=0, 0(with 5-HT), 0.5, 1, 1(with 5-HT), 2, 3, 8 are 7, 7, 10, 13, 8, 9, 18, 17, respectively. 5mins/animal. The experiments were repeated more than twice for the followings: OD_{600}=1, 3, 8.
Fig. 4C	The numbers of animals used are (from left to right) are 33, 18, 14, 43, 17, 44, 28, 18, 47, 17, 113, 15, respectively. 5mins/animal. The experiments were repeated more than twice for the followings: N2=4, HSN;NSM=3.
Fig. 5A	The number of animals used are as follows (in the order of increasing concentration): N2 (as in Fig. 2); *tph-1* (as in Fig. 4); *ser-1(ok345)* = 19, 23, 27, 23, 34, 18; *ser-1(opy027)* = 28, 44; *ser-4(ok512)* = 13, 19, 27, 21, 61, 16, 23; *ser-4(opy024)* = 21, 84; *ser-5* = 12, 29, 17, 16; *ser-7* = 16, 17, 32, 16, 14, 18; *mod-1* = 14, 17, 16, 20, 19; *ser-5;ser-4;mod-1;ser-7 ser-1* = 14, 18, 41, 23, 69. 5mins/animal.
Fig. 5B	The numbers of animals used are (from left to right) are 33, 18, 34, 42, 61, 84, 16, 14, 20, 69. 5mins/animal. The experiments were repeated more than twice for the followings: N2 =4, *ser-1(ok345)*=3, *ser-4(ok512)*=4, *ser-7*=3, *ser-5;ser-4;mod-1;ser-7 ser-1*=7.
Fig. 6A, B, C<br><br>Supp. Fig. 6D	The number of animals used are as follows (in the order of increasing concentration): N2= 62, 11, 20, 14, 18, 24; *ser-1(ok345)* = 19, 13, 13, 14; *ser-4(ok512)* = 13, 16, 15, 13, 13; *ser-4(opy024)* = 30, 15; *ser-5* = 12, 9, 13, 12; *ser-7* = 16, 7, 9, 15, 11; *mod-1* = 14, 9, 10, 13; *ser-5;ser-4;mod-1;ser-7 ser-1* = 14, 8, 7, 19, 11. 5mins/animal. The experiments were repeated more than twice for N2 (0mM)=4 and N2 (5mM)=3.
Fig. 6D, E	The numbers of animals used are (from left to right) are 18, 7, 14, 12, 13, 30, 12, 15, 10, 19. 5mins/animal. The experiments were repeated more than twice for the followings: N2 =3, *ser-7*=3, *ser-5;ser-4;mod-1;ser-7 ser-1*=3.
Supp. Fig. 4B	The numbers of animals used are (from left to right) are 113, 13, 13, 47, 17, 19, 10, 14, 24. 5mins/animal. The experiments were repeated more than twice for the followings: N2 =5, ADF(TeTx)=4, HSN;NSM=3.
Supp. Fig. 4G, H	The numbers of animals used are (from left to right) are 28, 20, 47, 30. 5mins/animal.
Supp. Fig. 4I	The numbers of animals used are (from left to right) are 28, 39, 47, 16. 5mins/animal.
Supp. Fig. 4J	The numbers of animals used are 9(N2), 13(NSM). 5mins/animal.
Supp. Fig. 5B	The numbers of animals used are (from left to right) are 64, 9, 23, 18, 21, 17, 16, 16, 23. 5mins/animal. The experiments were repeated more than twice for the followings: N2 =3, *ser-1(ok345)*=3.
Supp. Fig. 5E	The numbers of animals used are (from left to right) are 33, 14, 34, 25, 103, 84, 80. 5mins/animal. The experiments were repeated more than twice for the followings: N2=4

Supp. Fig. 6E, F	The numbers of animals used are (from left to right) are 18, 15, 14, 34, 30, 12. 5mins/animal.<br>The experiments were repeated more than twice for the followings: N2 =3, *ser-7*=3.
